# Defining ‘quality’ from the patient's perspective: findings from focus groups with Medicaid beneficiaries and implications for public reporting

**DOI:** 10.1111/hex.12466

**Published:** 2016-04-28

**Authors:** Ryan P. Theis, Jevetta C. Stanford, J. Robyn Goodman, Lisa L. Duke, Elizabeth A. Shenkman

**Affiliations:** ^1^ Department of Health Outcomes and PolicyCollege of MedicineInstitute for Child Health PolicyUniversity of FloridaGainesvilleFLUSA; ^2^ Department of PediatricsCollege of Medicine JacksonvilleUniversity of FloridaJacksonvilleFLUSA; ^3^ Department of AdvertisingCollege of Journalism and CommunicationsUniversity of FloridaGainesvilleFLUSA; ^4^ Department of Health Outcomes and PolicyCollege of MedicineUniversity of FloridaGainesvilleFLUSA

**Keywords:** health care quality, patient‐centred care, public insurance, public reporting, qualitative

## Abstract

**Background:**

With an increased emphasis on patient‐centred outcomes and research, investigators seek to understand aspects of health care that are most important to patients. Such information is essential for developing report cards that present health‐care quality information for consumers, which many states are adopting as a strategy to promote consumer choice.

**Objective:**

This study examined the processes that women in Medicaid follow for selecting health plans and explored their definitions of ‘good’ and ‘poor’ quality health care.

**Design:**

We conducted focus groups with Medicaid beneficiaries in four Texas communities, using quota sampling to ensure representation of different racial/ethnic, eligibility and geographic groups.

**Results:**

We conducted 22 focus groups with 102 participants between October 2012 and January 2013. In a free‐list exercise, ‘doctors’ represented the most important aspect of health care to participants, followed by cost, attention, coverage and respect. Discussions of health‐care quality revealed an even mix of structural factors (e.g. timeliness) and interpersonal factors (e.g. communication), although few differences were observed by beneficiary characteristics. Participants linked themes in their overall framing of ‘quality’ – revealing processes of care that affect health outcomes (e.g. discontinuity of care resulting from poor communication with providers) and which were often mediated by advocate providers who assisted patients experiencing barriers to services.

**Discussion and conclusions:**

Findings support other studies that highlight the importance of the patient–provider relationship. Patient‐centred definitions of health‐care quality can complement predominant provider‐centred conceptual frameworks and better inform initiatives for public reporting of quality measures in these populations.

## Introduction

The Institute of Medicine (IOM) identified patient‐centredness as one of six key factors for improving health‐care quality in the 21st century.^1^ Health care that is patient‐centred ‘is respectful of and responds to individual patient preferences, needs, and values’, ensures that patient values guide all health‐care decisions, and provides patients the education and support they need to participate in their own care.^1^, ^2^

In the United States, the Medicaid programme provides health‐care coverage for low‐income individuals (including children, pregnant women, parents, seniors and individuals with disabilities), and the Children's Health Insurance Program (CHIP) covers children whose family income is too high to qualify for Medicaid, but too low to afford private insurance. Both public insurance programmes are administered at the state level and have seen a widespread transition from fee‐for‐service to managed care delivery models, in which health plans contract with the states to provide services based on a capitated (per member per month) payment. Under managed care, Medicaid beneficiaries and parents of CHIP beneficiaries face numerous decisions about their coverage, including options on health plans and primary care providers available to them.^3^ Studies have found that Medicaid beneficiaries have more difficulty than privately insured consumers in knowing how to judge their health plan options, and often do not understand that their health plan is related to the care they receive.^3^

To assist beneficiaries in making these types of decisions, state agencies have increasingly turned to the use of report cards that compare providers and health plans.^4^, ^5^ For state Medicaid agencies, the public reporting of health plan performance is a policy option that can also help to incentivize quality improvement.^6^ In 2010, more than three‐fourths of states with Medicaid managed care publicly reported on the quality of their health plans, and 15 of these states prepared quality report cards that beneficiaries could use to compare and choose health plans.^7^

Studies have found mixed results on the effectiveness of health plan report cards in helping Medicaid members make informed decisions about which health plan to join.^3^, ^8^, ^9^, ^10^ This may occur because patients have no interest in using report cards, they cannot understand the content,^4^ or they feel the report cards do not reflect their definition of ‘quality’ health care. In these cases, ensuring the development of effective report cards means placing the patient at the centre of the process.

In Texas, Senate Bill 7 (82nd Legislature) requires the state Medicaid agency to develop report cards for new enrollees that provide comparative results on health‐care quality before they choose their health plans. At the time of this legislation, 18 different health plans participated in Texas Medicaid, and depending on their service area within the state, new enrollees had up to five health plans from which to choose. In the absence of report cards available to the public, these decisions were based largely on provider directories and word of mouth. This article presents findings from focus groups we conducted in Texas to better understand how women in Medicaid select health plans and to explore their perceptions of ‘good’ and ‘poor’ quality health care – findings that were later used to inform the development of health plan report cards for Texas Medicaid and CHIP.

## Methodology

Focus groups were conducted in October 2012 and January 2013 in four diverse Texas communities. Based on known disparities in health‐care quality by race/ethnicity^11^, ^12^, ^13^, ^14^, ^15^, ^16^ and rurality,^17^, ^18^ we used quota sampling to ensure representation of three racial/ethnic groups, urban and rural residents, and four eligibility groups. The eligibility quota represents adult members in Texas Medicaid managed care (STAR or STAR+PLUS) or parents of children enrolled in STAR or CHIP.^19^ The STAR+PLUS programme integrates acute and long‐term services for recipients of Social Security Income (SSI) and SSI‐related clients. STAR+PLUS members were divided into two quotas – those dually eligible for Medicaid and Medicare (the US federal public health insurance programme for individuals 65 years and older) and those only in Medicaid. Twenty‐four focus groups were planned, representing one group for each combination of characteristics. This design permits an overview of patient perceptions of health‐care quality in Medicaid, while allowing comparisons of findings by quota, with eight focus groups for each racial/ethnic category, 12 for each geographic category and six for each programme eligibility category. All urban groups were conducted in a major metropolitan area, while rural groups were conducted in three separate sites.

### Sample

The sample was drawn from enrolment data obtained by the Medicaid and CHIP health plans, and included all adult female beneficiaries (≥21 years) in STAR and STAR+PLUS, and all child and adolescent beneficiaries (≤18 years) in STAR and CHIP who lived in one of the study sites. Participation was restricted to female beneficiaries and caregivers of child beneficiaries because women represent 60% of non‐elderly adult Texas Medicaid enrollees^20^ and make approximately 80% of health‐care decisions for their families in the United States.^21^ This restriction is also a common strategy in focus groups to encourage disclosure with respect to gender.^22^ Residences of beneficiaries were mapped using ArcGIS, which optimized the matching of study sites with participants.

### Focus groups

Twenty‐two focus groups were conducted with 102 participants (Table 1). Participants ranged from 19 to 81 years old and all were women, with the exception of one man who accompanied a female participant. Two experienced moderators conducted each focus group, with a third bilingual moderator for Hispanic groups. Attendance ranged from 2 to 9 members, with a mean of 4.6 members per group. Discussions were audiotaped with the participants’ consent. Approval through the University of Florida Institutional Review Board was obtained for analysis of the focus group data.

**Table 1:** Characteristics of focus group participants

	*n*	%
Race/ethnicity		
Black, non‐Hispanic	35	34
Hispanic	43	42
White, non‐Hispanic	23	23
Other, non‐Hispanic	1	1
Programme eligibility		
STAR (Medicaid) adult	23	25
STAR (Medicaid)/CHIP caregiver	25	23
STAR+PLUS (Medicaid SSI)	27	27
STAR+PLUS (dual‐eligible)	27	27
Community		
Rural	59	58
Urban	43	42
Gender		
Female	101	99
Male	1	1
Age		
18–35	23	23
36–50	27	27
51–65	27	27
66+	10	10
Unknown	15	15

To inform the development of meaningful report cards, we asked participants how they define ‘quality’ health care, what aspects of care were most important to them, and what type of content would encourage them to use report cards. Report card mock‐ups were distributed to participants during the sessions.^23^ Each member received a gift card for participation.

### Analysis

Audiotapes of 21 sessions were transcribed and translated to English as appropriate, and transcripts were uploaded into Atlas.ti 7.5 for coding. The analytic approach followed iterations of content analysis and grounded theory,^22^, ^24^ with the primary phase focusing on known dimensions of health‐care quality (e.g. timeliness, communication). Additional codes were created through open coding to capture emergent themes.^25^

Two researchers independently coded each transcript, which were combined and analysed in team meetings to quantify inter‐rater reliability.^25^ Discrepancies were resolved by team consensus, with the assistance of a third researcher to resolve discrepancies that remained after group discussion. This methodology allowed for a final inter‐rater reliability of 100%.

The themes assessed in the primary phase were counted across all transcripts and by quota. The primary authors conducted a phase of secondary coding, using grounded theory to identify emergent themes in quotations with the most common primary codes. One focus group of six Black rural mothers was not audio‐recorded. This group is included in summaries of free‐list data, but not in the content or grounded theory analyses.

## Results

### Free‐list – ‘Most important things for getting good quality care’

During the focus groups, participants were asked to write two responses to the question: ‘When you think about getting good quality health care, what is most important to you?’ The majority provided more than two items (mean number of items = 2.46; range: 0–6), and the findings were later compiled, recoded into single‐item responses, and analysed as a free‐list.^22^

**Figure 1:**
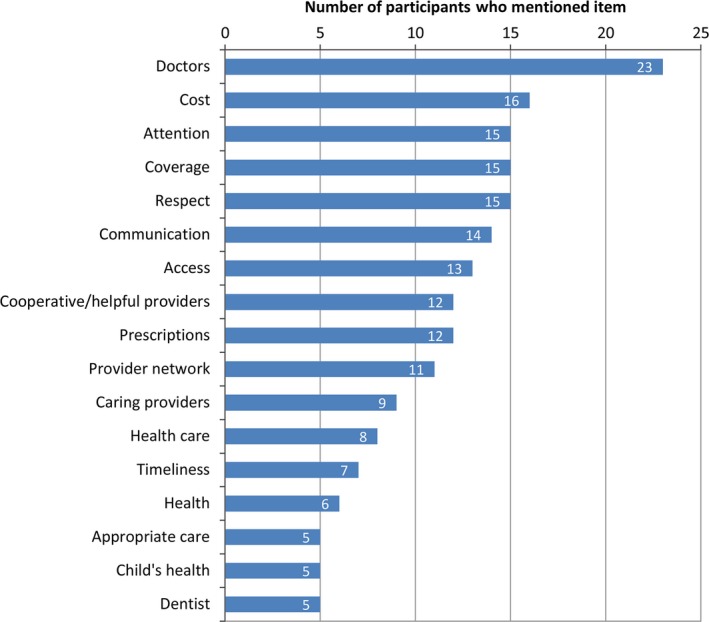
Free‐list: ‘Most important things for getting good quality health care’.

Figure 1 summarizes the ‘most important’ aspects of quality that were listed by five or more participants. Three types of responses were observed: (i) access to broad categories of health care, such as doctors or prescription drugs; (ii) aspects of health care that are *interpersonal*, such as having providers who are attentive; and (iii) aspects of health care that are *structural* (attributed to the health‐care system, rather than individuals), such as having affordable care. In addition, many participants listed their health or their child's health without reference to the health‐care system. The interpersonal/structural dichotomy is an important feature of models used in the social sciences for understanding experiences of discrimination^26^ and is consistent with the social ecological model used to study social determinants of health.^27^

For many participants, the most salient aspect of good quality care was simply having it. Twenty‐three participants listed ‘doctors’ without other qualifying terms. Twelve participants listed ‘medicine’, and five listed ‘dentists’. These types of responses reflect the scope of coverage available to Texas Medicaid and CHIP beneficiaries at the time of the study, the availability of provider types within health plan networks, or the extent and frequency of copays. However, without other qualifiers, such responses could not be grouped into other categories. It is possible, for example, that a participant's listing of ‘doctor’ refers not only to having a doctor, but also to the various qualities of doctors discussed below.

### Focus group discussion themes

A total of 16 ‘quality’ codes were included in the content analysis, which were divided into the interpersonal and structural categories that emerged in preliminary coding. Seven codes addressed interpersonal aspects – attentive care, communication, customer service, decision making, diagnosis, doctor respect and encounter time. Nine codes addressed structural aspects – after‐hours care, benefits, continuity, copays, coverage, information, provider networks, timeliness and transportation. These codes were further divided into themes related to ‘good quality’ and ‘poor quality’, as applicable. This allowed assessing members’ experiences with health care, their valuation of these experiences and how these experiences figure into members’ definitions of ‘quality health care’. It is important to note that few differences in findings were observed among the focus group quotas – race/ethnicity, geography and eligibility. Findings are therefore grouped by the main themes that emerged during coding; differences by quota are presented in categories where they were observed.

Table 2 lists the five most common themes within each of the two general categories of health‐care quality – interpersonal and structural – along with representative quotes from transcripts to illustrate each. Table 3 shows the number of quotations coded for each of these themes across all transcripts, and the distribution of ‘good’ and ‘poor’ quality codes within each theme.

**Table 2:** Themes related to good and poor health‐care quality among Medicaid beneficiaries

Theme and coded definition	Representative quote
Interpersonal themes	
*Attentiveness*: Remarks on how the provider pays adequate attention to the patient's needs, related to both personal and medical needs	‘… they [providers] listening to what I'm telling them and are they open to things that I want to implement as far as my medications or health care or just anything that I want them to talk with me about’. – *Black rural STAR*+*PLUS beneficiary (dual‐eligible)*
*Communication*: Comments on the quality of communication with providers or staff in the provider's office(s)	‘When I'm talkin’ to my doctor, is he listening, does he know my fears, does he understand what my needs are when I leave him, what my concerns are?’ – *Black urban STAR*+*PLUS beneficiary (Medicaid‐only)*
*Provider Respect*: Remarks about the presence or absence of respectful treatment from providers and office staff	‘I think this might sound old‐fashioned, but bed side manners, the way the doctor talks to you, treats you. That means a lot to me. I mean, it's a big deal’. – *White urban parent (STAR/CHIP)*
*Diagnosis*: Comments about quality of the diagnosis; remarks about the procedures performed to arrive at the diagnosis and prescribing medications to treat or manage a condition	‘Yes, I think you're going to take the medication and later you're going to do an analysis. Every three months they tell you, “Let's do the analysis to see if the medication is working for you,” not just for high blood pressure but for diabetic[s], for anything’. –*Hispanic rural STAR*+*PLUS beneficiary (dual‐eligible)*
*Encounter Time*: Comments related to the amount of time providers spend with patients during the medical examination/office visit	‘… The doctor isn't really trying to get to your issue. They just want to see you and out, you know. ‘I have 20 minutes to spend with each patient, and you're taking too much of my time, “cause I got other patients to see”’. – *Black rural STAR beneficiary*
Structural themes	
*Benefits*: Remarks related to health plan coverage of benefits, such as dental and vision; comments about access to (or lack of) services, such as medications. Includes comments about having to pay for non‐covered benefits	‘The [health plan] that I have now … is a world of difference, because I couldn't get therapy before. I get it now. I couldn't go to the dentist before. I can go now. I couldn't get transportation before, and I can get it now’. – *Black urban STAR*+*PLUS beneficiary (dual‐eligible)*
*Copay*: Comments about out‐of‐pocket expenses or copays, both the presence and absence of	‘I have not had to put a dime out since I've actually been on Medicaid. Not a penny's come out of my pocket for my medical problems’. – *White rural STAR*+*PLUS beneficiary (Medicaid‐only)*
*Network*: Remarks about the health plan because of the quality and availability of providers within its network	‘It took me a little bit of calling around through [the health plan] to find out if he was a part of [health plan's] group. That was my major thing, because if he was not, then it would have entailed some very anxious moments for me’. – *Black urban STAR*+*PLUS beneficiary (Medicaid‐only)*
*Timeliness*: Remarks about wait times at the office, as well as time spent waiting for scheduled appointments	‘I haven't had any problems. I've been able to just call and say, “Okay, I need an appointment.” And they say, “Well okay, come in the day after tomorrow …”’ – *Black urban STAR*+*PLUS beneficiary (Medicaid‐only)*
*Continuity of Care*: Remarks about receiving continuous care, which includes seeing the same providers on each visit and getting follow‐up care	‘I have sent in two foot doctors. Now I'm on my third foot doctor because they didn't want to pay for it … And I guess they don't want to pay so they keep pushing me off to different doctors’. – *Black urban STAR*+*PLUS beneficiary (dual‐eligible)*

**Table 3:** Interpersonal and structural aspects of health‐care quality – content analysis by valuation

	Number of quotations (in all focus group transcripts)				
	Total	Good		Poor	
	*n*	*n*	%	*n*	%
Interpersonal themes					
Provider respect	83	48	58	35	42
Communication	73	41	56	32	44
Diagnosis	73	41	56	32	44
Attentiveness	58	34	59	24	41
Encounter time	33	14	42	19	58
Structural themes					
Benefits	113	66	58	47	42
Timeliness	88	38	43	50	57
Network	68	36	53	32	47
Copay	63	36	57	27	43
Continuity	49	27	55	22	45

### Interpersonal quality of care

#### Provider respect

Provider respect was the most frequently mentioned interpersonal theme, emerging in all but three focus groups. Participants focused on personal treatment by providers (or lack thereof) and the desire to be treated like a person. More specifically, participants emphasized respectful interactions with providers and office staff during visits. Patients provide a more comprehensive and transparent description of symptoms when addressed respectfully by providers. One participant expounded on how provider respect can influence disclosure by patients:

You do feel more secure with a doctor who seems to have a proactive attitude with regard to your care, because he's sending an empathic connection to you, letting you know that he does care. And because he cares, then you feel more apt to give him more information and allow him an opportunity to help you, especially in a situation where you may find yourself in a position where you can't help yourself. –Black urban STAR+PLUS beneficiary (Medicaid‐only)

#### Communication

Communication and diagnosis were tied for the second most frequently mentioned interpersonal theme. Communication was discussed in all but four focus groups, while diagnosis was discussed in all focus groups. Participants emphasized the importance of receiving clear lay explanations from providers of their health conditions and proposed treatments. Additionally, participants valued receiving information from providers that takes into account specific personal, social or environmental factors. Participants expressed a preference for dialogue rather than one‐sided encounters where providers talk ‘to’ or ‘at’ them.

Yeah, they spend time and they talk to me. Like sometimes I don't understand. They take the time to tell me and sometimes I ask them but I still don't understand. They'll go to my daughter and talk to my daughter and explain it to her so that she can tell me. – Hispanic urban STAR+PLUS beneficiary (Medicaid‐only)

#### Diagnosis

Participants emphasized the value of receiving a definitive diagnosis from providers when raising health concerns during a visit. Many expected encounters to resolve with a diagnosis without needing referral to a specialist, and participants expressed frustration with confirmatory diagnostic testing, especially when the process did not result in a conclusive diagnosis. Participants described poor treatment outcomes resulting from incorrect diagnoses, which they often attributed to factors such as poor coordination among multiple providers or provider carelessness. One participant described having to go ‘back and forth’ between urgent and primary care settings to receive a diagnosis and treatment for her child:

I just told his doctor yesterday that he's been running a little fever and he seems like he's having a hard time breathing. She said she didn't hear anything in his lungs, but she wasn't qualified to make any diagnosis. The next time I hear him breathing funny, take him to the emergency room and let them do x‐rays … Then the emergency room tell you to take “em to your doctor”. – Black urban parent (STAR/CHIP)

#### Attentiveness

Participants often described and attributed value to the attentiveness of providers, focusing on the attention providers pay to patients when providing care. Attentiveness was a topic of discussion in 16 of the 21 recorded focus groups. Participants valued providers who listen to and genuinely take into consideration their expressed concerns, suggestions or information related to their medical history, and who provide a thorough examination during the visit.

#### Encounter time

Encounter time was the fifth most described interpersonal quality theme, noted in more than half of the focus groups (12 of 21), and referring to the length of time the provider spends conducting the actual examination. Quotations about encounter time often had relevance to provider attentiveness and communication, as participants stressed the importance of providers not only taking adequate time during the visit, but also taking time to explain diagnosis and treatment plans. Participants generally ascribed more value to encounters of longer duration. One participant described short encounter time as an indicator of poor service:

I guess the way they treat you, the staff, how much time they spend with you examining you in the room. Because if they're just going to spend two minutes and say, “Well, this..” and then just leave, then that's not a very good doctor … If they're just going to check you and say, “We'll be right back,” then that's not good service. You can tell. – Hispanic rural parent (STAR/CHIP)

### Structural quality of care

#### Benefits

Benefits were the most common structural theme in the study and emerged in all focus groups. Comments about benefits dealt with a wide range of services – most often dental, vision and prescription benefits – but also including specialist, preventive, and perinatal care, special therapies, adaptive aids, and transportation services. Adult participants described insufficient coverage for dental care and vision care. Some participants related situations where prescription benefits were switched after joining their health plan. One expressed a sense of disempowerment on learning that her new plan would not cover her medications for osteoporosis:

Well until you're in it, that's when you realize what they're going to give you and what they're not going to give you. I had [drug plan A] until the year 2012. Well, then they tell me they're going to switch me to [drug plan B]. Now he's sending me letters saying there are medicines I have that it's not going to provide for me. So now that's what I have to know … who I can turn to give me the medicines I need. – Hispanic rural STAR+PLUS beneficiary (dual‐eligible)

#### Timeliness

The second most common structural theme was timeliness of care – both in terms of appointments with providers and waiting to be seen at the provider's office. Timeliness was discussed in all but four focus groups. Discussions about time spent in the provider office or emergency department waiting room were the most salient. In regard to primary care, timeliness was often a key factor determining a beneficiary's selection of a primary care provider. For one participant, long wait times in the office led her to leave a personal doctor whom she otherwise held in high regard:

I was going to [doctor] and he was really careful and you know … But it was just the time. You know, sittin’ there looking at the TV. But when you do see him, he's very caring to you. He listening at you, you know? … But I left him … I went to another doctor to try to get that time. – Black urban STAR+PLUS beneficiary (Medicaid‐only)

In two Hispanic focus groups, timeliness was discussed in terms of appointment availability. Getting themselves and their children into the doctor's office was often a matter of finding providers who were open on weekends or after hours. Some participants adopted a strategy of seeing providers who did not make appointments, such as urgent care centres.

#### Provider network

Remarks about health plans because of the quality or availability of network providers comprised the third most common structural theme and were present in all but one focus group. These discussions included complaints about a plan not having a particular physician, a physician no longer taking the plan or taking new patients, or a plan assigning the participant to a new physician. This category also included comments about the location of providers’ offices – both in terms of proximity to participants and the quality of the neighbourhood.

An important topic in this theme was the assignment of providers to beneficiaries upon joining Medicaid or their health plan. Participants reported negative experiences with provider assignment upon joining a health plan, particularly when they already had a provider with whom they had a good rapport. In some situations, this led them to retain out‐of‐network providers – accepting the copays to keep their preferred providers. Adults in Medicaid and the STAR+PLUS programme were more likely to stress the importance of choice of specialists who could care for specific chronic conditions, such as diabetes.

#### Copay

Having low copays for health care was the fourth most frequently mentioned structural theme and was mentioned in all but four focus groups. Participants’ experiences with copays were good overall, revealing a pattern of high satisfaction with dental, vision and prescription copays. Some participants described lower copays following a switch in coverage – particularly in regard to prescription medication. In three focus groups, participants had positive experiences receiving assistance with copays from the programme, health plan or provider. As in other areas of health service delivery addressed in this study, participants expressed greater satisfaction with providers who are proactive, who advocate for their patients and who ‘work’ with patients when they cannot afford out‐of‐pocket expenses.

#### Continuity

Discussions on continuity of care represented the fifth most common structural theme, mentioned in 15 focus groups. A salient concern for enrollees was ensuring they would still have their personal doctor after joining their health plan. In one group, participants stated that being able to retain their personal doctors would be a factor in their decision to keep or switch their plans. Participants also valued keeping doctors specifically because they know their medical history. This was especially true for STAR+PLUS participants, who have greater need of care for chronic conditions and disabilities, and therefore have more complex medical histories. Participants whose doctors knew their medical histories were more confident in their doctors’ ability make the right treatment decisions. One member with vision impairment described the process of selecting new doctors as ‘overwhelming’.

It's not a matter of complacency with me, because I had an opportunity to go to the new doctor and try to see what he would do … And then I thought, “Oh Lord, developing a whole new relationship.” Jesus … You know, like some of my other doctors are they going to know my prescription? Are they going to know that I really can't see out of this eye here? … I'm wondering if these doctors, do they know my current medical record? – Black urban STAR+PLUS beneficiary (Medicaid‐only)

## Discussion

Findings from this study highlight the most salient elements of a patient‐centred framework for health‐care quality, which is essential for developing effective tools for public reporting of health‐care quality results. This study supports other work that has shown Medicaid members prefer to base health‐care decisions on the testimony of an individual plan member than on aggregated data.^28^ Participants in this study stressed the importance of good communication with providers, including receiving clear explanations and respectful treatment, which reinforces findings in the literature on the importance of the patient–provider relationship^29^, ^30^, ^31^ and for including physician communication and encounter time as measures of quality in public reporting.^3^ Provider communication was therefore identified as a key indicator to include on health plan report cards produced for Texas Medicaid.^32^ This is consistent with measure sets used in other consumer‐oriented state Medicaid report cards such as those produced by Maryland^33^ and Michigan,^34^ which use some or all items that comprise the Consumer Assessment of Healthcare Providers and Systems (CAHPS^®^) *How Well Doctors Communicate* composite measure.^35^

The findings from this study also support the inclusion of a measure that addresses provider respect, which is typically not included on publicly available state Medicaid report cards – except when implicitly part of the CAHPS^®^ composite. (One item in the CAHPS^®^ *How Well Doctors Communicate* composite asks respondents: ‘In the last six months, how often did your personal doctor show respect for what you had to say?’) Furthermore, the general emphasis on the importance of personal doctors in this study suggests that a patient‐reported personal doctor rating is important.

In regard to structural aspects of care, information on benefits, provider networks and copays is generally included in other types of materials (e.g. member handbooks) that are sent to new enrollees. Timeliness of care can be addressed in report cards using self‐report measures, as seen in report cards produced by Medicaid programmes in California^36^ and Colorado,^37^ and was considered a key indicator on report cards for Texas Medicaid.

Few differences in beneficiary perceptions of interpersonal health‐care quality were observed by participant race/ethnicity, geography or programme eligibility. The study revealed some differences in the structural domains – with Hispanic groups more likely to reveal a preference for using urgent care centres for primary care, and STAR+PLUS (SSI‐eligible) groups placing greater importance on the availability of specialist care and of personal doctors who know their medical histories.

Certain limitations of this study may affect inferences that can be made to Medicaid and CHIP populations generally. First, the study findings are specific to Texas and may not be generalizable to other states. Second, because only women were included in the study, findings may not be generalizable to men enrolled in Medicaid. Although one focus group included a man, this was a deviation from the study protocol. We did not consider the inclusion of a male participant to have provided sufficient representation of male perspectives; at the same time, we did not observe his participation to have affected disclosure among women in the focus group. Third, the study lacked full representation of STAR+PLUS beneficiaries, as two focus groups in this quota were cancelled due to problems with the facility. However, this study achieved saturation with the majority of eligibility groups in Texas Medicaid and CHIP, and focus groups with disabled beneficiaries are planned for future study.

The enrolment data used to select participants did not always reflect participants’ self‐report of race/ethnicity. Four adult focus groups each had one participant who self‐identified as belonging to a different racial/ethnic group than the others. Furthermore, participants were assigned to caregiver groups based on the child's race/ethnicity, as only enrolment data for children were available during sampling. In two cases, discordance between the child's and parent's racial/ethnic group resulted in mixed racial/ethnic composition of caregiver focus groups. These six focus groups were excluded from analyses to compare findings by race/ethnicity.

## Conclusion

Patient‐centred frameworks for health‐care quality can be used to complement existing health‐care quality frameworks and to guide initiatives for public reporting. In addition to revealing (or confirming) those aspects of health care that are most important to Medicaid beneficiaries – respect and communication from doctors, ample benefits and timely care – the focus groups showed ways in which beneficiaries perceive domains in relation to one another.

Continuity of care was essential for good outcomes and in many cases was influenced by the interpersonal qualities of providers. Respect and attentiveness from providers leads to better communication in clinical encounters, which in turn leads to better patient confidence in providers’ diagnoses and more equitable decision making for treatment. Timeliness of care was often an important factor in beneficiaries’ choice of providers – in some cases leading beneficiaries to stay with providers they perceived to be of ‘lower quality’ or incur greater out‐of‐pocket expenses.

Patient preferences and perceptions of the quality of their care are critical for informing the selection of health‐care measures for public reporting and the manner in which measures are presented. Qualitative work such as that presented in this article is essential for ensuring that report cards and other public reporting initiatives are patient‐centred and therefore more likely to be used by their target populations.

## Conflict of interests

The authors report no conflict of interests.

## Source of funding

This work was funded by an External Quality Review Organization (EQRO) contract with the State of Texas Health and Human Services Commission, United States.
